# Daycare-simulated interrupted phototherapy for neonatal jaundice: a randomized controlled trial

**DOI:** 10.1007/s00431-025-06727-z

**Published:** 2026-01-10

**Authors:** Jia Cheng Ong, Farohah Che Mat Zain, Yee Cheng Kueh, Noraida Ramli, Hans Van Rostenberghe, Surini Yusoff

**Affiliations:** 1 Department of Paediatrics, School of Medical Sciences, Universiti Sains Malaysia, Kota Bharu, Kelantan 16150 Malaysia; 2 Hospital Pakar Universiti Sains Malaysia, Kota Bharu, Kelantan 16150 Malaysia; 3 Faculty of Medicine, Universiti Sultan Zainal Abidin, Medical Campus, Kuala Terengganu, 20400 Malaysia; 4 Biostatistics & Research Methodology Unit, School of Medical Sciences, Universiti Sains Malaysia, Kota Bharu, Kelantan 16150 Malaysia; 5 International Medical School, Management and Science University, Shah Alam, Selangor 40000 Malaysia

**Keywords:** Continuous phototherapy, Neonatal jaundice, Daycare, Interrupted phototherapy

## Abstract

The aim of the study was to compare the effectiveness of daycare-simulated interrupted phototherapy versus continuous phototherapy for treating neonatal jaundice. A parallel randomized controlled trial with one-to-one allocation was conducted involving low-risk jaundiced neonates. The neonates in the intervention group received 10 h of phototherapy, while the control group received continuous phototherapy for 24 h. Total serum bilirubin (TSB) was measured before the start of phototherapy and at 24 h of treatment. Seventy-four neonates were recruited, and 37 neonates were randomly allocated to each group. The mean rate of fall of TSB per hour was not statistically significantly different between the intervention and control groups (1.71 versus 1.9 µmol/L/h; *p* = 0.529). The mean of TSB post-treatment in the intervention group was higher than in the control group and statistically significant (182 versus 158 µmol/L; *p* = 0.045) but not clinically significant, as none of the neonates required reinstitution or continuation of phototherapy.

*Conclusion*: Ten hours of phototherapy which could be given in daycare may be effective and safe.

*Trial registration*: This trial wasregistered with Australian New Zealand Clinical Trials (trial ID: ANZCTR 12624000860561) prospectively on 12 July 2024.

**Table:** 

**What is Known:** • *Intermittent phototherapy is known to be as effective as continuous phototherapy.* **What is New:** • *We designed a study in which phototherapy was interrupted after 10 h, simulating daycare phototherapy.* • *Daycare phototherapy for 10 h may be feasible in treating neonatal hyperbilirubinemia in low-risk neonates.*

**What is Known:**

• *Intermittent phototherapy is known to be as effective as continuous phototherapy.*

**What is New:**

• *We designed a study in which phototherapy was interrupted after 10 h, simulating daycare phototherapy.*

• *Daycare phototherapy for 10 h may be feasible in treating neonatal hyperbilirubinemia in low-risk neonates.*

## Introduction

Phototherapy is the mainstay of treatment for neonatal jaundice since the 1950s. It involves rapid photoisomerization of bilirubin resulting in faster excretion [1]. Traditionally, phototherapy is given until the serum bilirubin level reaches safe thresholds to avoid bilirubin neurotoxicity even in low-risk neonates. This practice disrupts the normal breastfeeding behaviour, may separate the child from the mother, causes burden to the hospital staff, and increases healthcare costs.

Over the years, many studies were carried out to determine the optimal treatment in terms of safety, effectiveness, and duration of phototherapy in treating neonatal jaundice. The first research available in the literature regarding intermittent phototherapy was done in 1973 by Maurer et al. [2]. Till date, there are various types of intermittent phototherapy carried out [3, 4]. Reviewing all the studies, there is still no definite treatment duration of phototherapy in neonatal hyperbilirubinemia, and hence, the feasibility of daycare phototherapy is still debatable.

In this trial, the concept of interrupted therapy was used to determine the feasibility of practicing daycare phototherapy among low-risk neonates. The objective of this study was to determine the effectiveness and safety of daycare-simulated interrupted phototherapy (intervention group) in low-risk neonates compared to continuous phototherapy (control group).

## Methods

This is a parallel randomized controlled trial with one-to-one allocation, conducted in the special care nursery of Hospital Pakar Universiti Sains Malaysia, a teaching hospital located in the northeast of Peninsular Malaysia. This study was conducted from July 2024 to March 2025. This trial was registered with the Australian New Zealand Clinical Trials Registry (trial ID: ANZCTR 12624000860561) prospectively. No changes were made to the protocol after registration.

The inclusion criteria were neonates aged between 24 h and 2 weeks after birth, gestational age more than 38 weeks, and birth weight more than 2.5 kg. TSB should not be more than 50 µmol/L above the phototherapy level according to age-based criteria as determined by Malaysia Clinical Practice Guidelines of Neonatal Jaundice. The exclusion criteria were severe jaundice requiring intensive phototherapy and having risk factors such as ABO incompatibility, G6PD deficiency, clinical sepsis, rhesus isoimmunization, and syndromic neonates. Neonates who developed other medical problems such as sepsis, dehydration, or TSB rising to levels requiring intensive phototherapy after being included in the study were withdrawn.

The intervention group received 10 h of phototherapy followed by 14 h of discontinuation. To ensure safety for the intervention group, another bilirubin level from a venous blood gas sample was taken after 6 h of stopping phototherapy. If that bilirubin level reached the threshold level, the neonate was withdrawn from the study and given standard treatment.

The control group received 24 h of phototherapy without interruption. TSB levels were taken pre-treatment (0 h) and at 24 h after the start of phototherapy for all neonates. The decision to stop or continue phototherapy was based on TSB level at 24 h for both groups.

The phototherapy machines used were Babyblue LED phototherapy® (Turkey) with intensity at 15–30 µW/cm^2^/nm for both groups. The irradiance was checked using a photometer prior to the start of the phototherapy. All neonates received a similar feeding regimen and nursing care. They were given 3 hourly oral feeding, and diapers were changed every 3 h. Bathing time was within 30 min for each neonate daily.

Primary outcomes included the mean rate of fall of bilirubin per hour and mean difference of TSB levels between 0 and 24 h in both groups. Secondary outcomes included the incidence of treatment failure or complications of phototherapy in both groups.

The sample size was estimated using G*Power with a moderate effect size of 0.30 (based on expert opinion), an alpha value of 0.05, and a power of 0.80 with two groups and two time measurements. The sample size was estimated as 68 (34 in each group). After adding a 10% dropout rate, the estimated sample size was 37 per group. The sampling method was convenience-based. Neonates included in the study were randomized into two groups. Randomization was performed by a person not involved in the care of the neonates based on a computer-generated table of random numbers using blocks of various sizes to ensure equal numbers in both groups. The concealment of allocation was ensured by the use of sequentially numbered, sealed, and opaque envelopes. Blinding of the staff taking care of the neonates was impossible because of the nature of the intervention. However, the measurement of the outcome was an objective assessment of TSB levels.

All results were analyzed using Statistical Package for the Social Sciences (SPSS) programme for Windows version 30. The demographic data and numbers of treatment failure are presented in frequency and percentage. The rate of fall of TSB per hour was calculated for both groups after reviewing the pre and post-treatment TSB levels. Continuous variables were analyzed using independent *T*-test. Dichotomous variables were analyzed using Chi-square or Fisher’s exact test. Independent *T*-test was used to analyze the mean of TSB post-treatment among two groups.

## Results

**Fig. 1:**
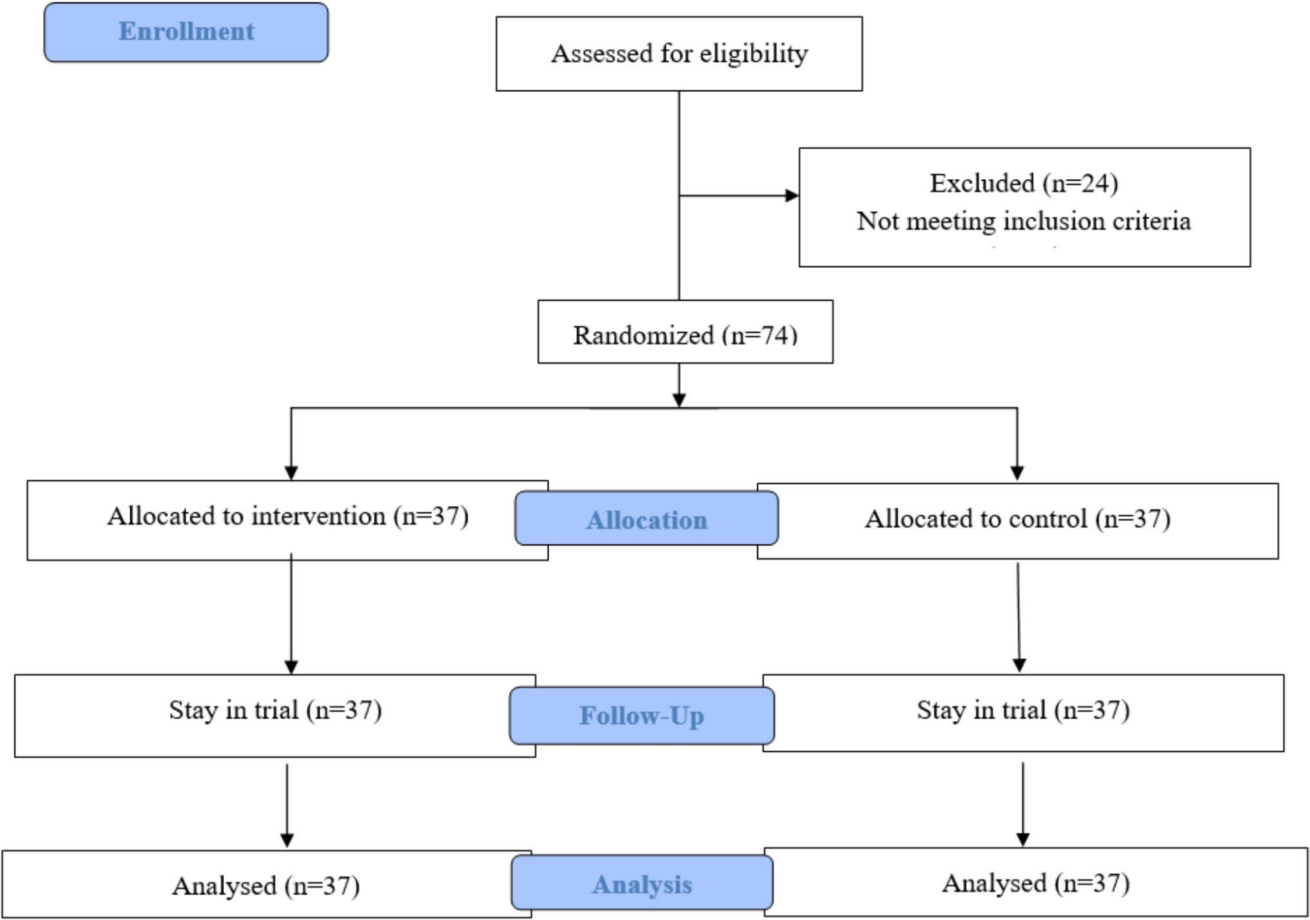
Participants’ allocation diagram

As shown in Fig. 1, a total of 98 neonates were assessed for eligibility. Seventy-four neonates fulfilled the inclusion criteria, and 24 neonates were excluded as TSB did not reach the threshold level. There were 37 neonates allocated to each group.

Table 1 shows the comparison of the baseline data between the two groups. There was no significant difference between the groups for weight, gender and gestational age but the neonates in the intervention group had higher postnatal age and higher TSB levels prior to starting phototherapy.

**Table 1:** Comparison of baseline variables and demographic data

Variables		Mean (SD)		t (df)	*p value*
		Intervention	Control		
Age at start of phototherapy (hours)		59.00 (20.19)	49.97 (12.64)	− 2.30 (72)	0.024*
Gestational age (weeks) Birth weight (kg)		38.65 (0.78) 3.05 (0.28)	38.7 (0.77) 3.06 (0.37)	0.297 (72) 0.139 (72)	0.767* 0.890*
Pre bilirubin (µmol/L)		224.16 (45.12)	204.22 (34.18)	− 2.14 (72)	0.018*
Gender^@^	Male	18 (48%)	18 (48%)	0.00 (1)	1.00^#^
	Female	19 (52%)	19 (52%)		

^@^Frequency (percentage) is reported

*Independent *T* test

^#^Chi-square test

Table 2 shows the mean rate of fall of TSB levels per hour was higher in the control group (1.90 ± 1.28 µmol/L/h) compared to the intervention group (1.71 ± 1.38 µmol/L/h); however, the mean difference between the two groups was not statistically significant (*p* = 0.529).

**Table 2:** Rate of fall of TSB per hour (µmol/L/h) and mean TSB levels post-treatment (µmol/L)

	Intervention group	Control group	*p* value
Mean rate of fall of TSB per hour (CI)	1.71 (1.24–2.17)	1.90 (1.47–2.33)	0.529*
Mean TSB levels post-treatment (CI)	182.30 (174.22–190.38)	158.27 (148.34–168.20)	0.046*

*Independent *T*-test

Table 2 also shows the mean difference of TSB levels post-treatment among the two groups. The TSB level in the intervention group was higher than in the control group, and this was statistically significant (*p* = 0.046).

No neonates in both groups experienced treatment failure, required another course of phototherapy, underwent double volume exchange transfusion, and required readmission for phototherapy. Four neonates, belonging to the intervention group, had small increases in TSB levels but did not require reinstitution of phototherapy.

## Discussion

This study suggested that 10 h of phototherapy had a similar success rate as a longer duration of phototherapy (24 h). The difference in the average rate of decline in TSB levels per hour was not statistically significant. The TSB levels at 24 h after starting phototherapy were significantly higher in the group receiving a short duration of phototherapy, but none of the neonates required reinstitution of phototherapy. This is because bilirubin levels increase with age corresponding with phototherapy thresholds. No complications of phototherapy were detected.

After the first course of 10 h of phototherapy, no neonate required reinstitution of phototherapy. Similarly, in the control group, no neonate required continuation of phototherapy. This was most likely due to strict selection criteria, including only neonates belonging to the low-risk group as in the Malaysia Clinical Practice Guideline for neonatal jaundice [5]. Hence, this study supports that intermittent phototherapy is feasible in low-risk neonates.

There were two systematic reviews performed on intermittent phototherapy versus continuous phototherapy for neonatal jaundice. Both reviews showed no significant difference in treatment effectiveness, adverse reactions, and decreases in serum bilirubin between continuous and intermittent phototherapy [3, 4]. Each review reported high heterogeneity between the trials. Some trials used intermittent phototherapy consisting of 1 h on followed by 2 h off for two cycles, while others used intermittent phototherapy for 12 h [1, 6]. This is the first study testing phototherapy of 10 h, which can be an appropriate duration for daycare practice.

In this study, there were four neonates in the intervention group who had slightly higher TSB levels 24 h after the start of phototherapy. This could be explained as mild rebounds of bilirubin levels after stopping phototherapy. The bilirubin may continue to increase concomitant with immaturity of the bilirubin conjugation process [7]. This increment of TSB was smaller than the increase in the phototherapy cutoff value according to the age. However, it did not require reinstitution of phototherapy. A proper continued monitoring for further rebound hyperbilirubinemia is necessary.

A limitation of the study was the statistically significant difference in the baseline characteristics such as age at start of phototherapy and baseline TSB levels. After proper randomization as performed in this study, one would expect that the difference in baseline characteristics (known and unknown factors) may balance each other and not affect the internal validity of the study. We recommend that the age of the neonates be standardized to reduce heterogeneity in the study.

## Conclusion

The result of this study shows that short phototherapy for 10 h may be effective and safe in treating neonatal hyperbilirubinemia in low-risk neonates. Due to the shorter duration required for treatment, this study model for daycare phototherapy can be an alternative option in order to reduce the number of admissions, potential mother–baby separation, breastfeeding failure, and healthcare costs.

## Data Availability

The data that support the findings of this study are not publicly available due to privacy and ethical restrictions involving study participants.

## Abbreviations

CI: Confidence interval
G6PD: Glucose-6-phosphate dehydrogenase
LED: Light-emitting diode
SD: Standard Deviation
SPSS: Statistical Package for the Social Sciences
TSB: Total serum bilirubin
